# Gradient Solvent Replacement‐Mediated Formation of High‐Strength Hydrogel‐Forming Microneedle for Long‐Term Drug Delivery

**DOI:** 10.1002/advs.202500833

**Published:** 2025-05-02

**Authors:** Hui Li, Fengzhen Meng, Chengwei Hu, Zhiyun Wu, Liuzhi Hao, Caijun Sun, Lijing Fang, Fan Pan, Shaoquan Bian, Huipeng Li, Mingjun Li, Bo Liu, Xiaoli Zhao

**Affiliations:** ^1^ Institute of Biomedicine and Biotechnology Shenzhen Institute of Advanced Technology Chinese Academy of Sciences Shenzhen 518055 P. R China; ^2^ Institute of clinical translation and regenerative medicine People's Hospital of Baoan District The Second Affiliated Hospital of Shenzhen University Shenzhen 518101 P. R China; ^3^ University of Chinese Academy of Sciences Beijing 100049 P. R China; ^4^ School of Public Health (Shenzhen) Sun Yat‐sen University Shenzhen 518107 P. R China; ^5^ Hebei Key Laboratory of Biomaterials and Smart Theranostics School of Health Sciences and Biomedical Engineering Hebei University of Technology Tianjin 300130 P. R China

**Keywords:** hydrogel‐forming microneedles, gradient solvent replacement, high‐strength, drug delivery, minimally invasive

## Abstract

The microneedle, a minimally invasive transdermal system, provides a convenient and painless method for drug delivery. Among the various types of microneedles, hydrogel‐forming microneedles (HFMs) demonstrate distinct advantages in terms of high‐dose drug loading and biocompatibility. However, HFMs usually require drying to obtain sufficient puncture strength, which may destroy drug activity and increase storage costs. Herein, a high‐strength HFM patch with pH‐responsiveness for post‐drug loading and long‐term release is developed based on acrylonitrile‐acrylic acid copolymer. The dipole‐dipole and hydrogen bonding interactions formed through gradient solvent replacement are evenly distributed within the cross‐linked network, significantly enhancing the mechanical properties of the hydrogel required for epidermal penetration. The prepared hydrogel exhibits a tensile strength of 26 MPa and a Young's modulus of 407 MPa. The microneedles formed from this hydrogel display a single needle mechanical force of 1.18 N. The post‐loading mode conferred by pH responsiveness allows the drug to be encapsulated in both the tips and the substrate, acting as a reservoir. Once applied to the skin, the microneedle is activated by body fluids to achieve long‐term drug release. Overall, this high‐strength HFM improves the mechanical properties in the hydrated state, making it a promising minimally invasive transdermal delivery platform.

## Graphical abstract

A high‐strength pH‐responsive hydrogel‐forming microneedle integrates gradient solvent replacement to enhance the mechanical properties of the acrylonitrile‐acrylic acid copolymer network, ensuring robust transdermal penetration and long‐term drug release. The system overcomes the limitations of existing hydrogel‐forming microneedles by maintaining strength in hydrated conditions and providing precise, pH‐triggered drug delivery for improved biomedical applications.

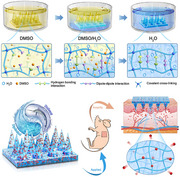

## 1 Introduction

Microneedle (MN) technology, a minimally invasive transdermal drug delivery system, provides a convenient and painless method for drug penetration.^[^ ^1^ ^]^ Unlike oral administration and subcutaneous injection, microneedles (MNs) circumvent issues such as gastrointestinal degradation and first‐pass metabolism.^[^ ^2^ ^]^ This technology also offers unparalleled advantages in convenient medication administration by alleviating pain, reducing the need for skilled administration, and minimizing the risks associated with hazardous waste disposal.^[^ ^3^ ^]^ With their unique ability to create mechanical microchannels, MNs provide a novel drug delivery method for drugs that traditionally require intravenous injection, such as nanomedicine, proteins, and nucleic acids.^[^ ^4^ ^]^ This has paved the way for novel approaches in treating a range of diseases.^[^ ^5^ ^]^

Differentiated by the structural design and mechanism of action, MN technology encompasses a variety of types, such as solid MNs, hollow MNs, coated MNs, dissolving MNs, and hydrogel‐forming microneedles (HFMs).^[^ ^6^ ^]^ Among them, HFMs have received significant attention since they were first reported in 2012.^[^ ^7^ ^]^ HFMs are constructed from swellable polymers that form hydrogel networks through cross‐linking between polymer chains, which facilitates high‐dose drug loading and sustained drug release.^[^ ^8^ ^]^ Furthermore, HFMs are biocompatible and can be fully removed post‐administration, thereby eliminating concerns about polymer residues in the skin.^[^ ^9^ ^]^ By modulating the structure of the hydrogel network, HFMs are particularly suitable for treating chronic diseases.^[^ ^10^ ^]^ For example, McGuckin et al. compared the ability of dissolving MNs and HFMs to release pramipexole (PRA) for Parkinson's in Sprague‐Dawley rats. Due to the rapid dissolution of the dissolving MNs, the drug delivery microchannels on the rat skin largely disappeared after 24 h of MN application. While the HFMs group exhibited the best long‐term drug release profile, with the highest PRA plasma concentration after 5 days of application.^[^ ^11^ ^]^

Despite these advantages, HFMs encounter substantial challenges in practical applications. The primary obstacle is their weak mechanical strength in the hydrated state, which hinders their ability to achieve optimal transdermal penetration. To improve the penetration ability, harsh treatments such as UV curing, long drying times are typically required before transdermal administration, which may destroy the activity of the encapsulated drug and increase the storage costs.^[^ ^12^ ^]^ In addition, the dry state HFMs swell dramatically after piercing the skin, which is not desirable for the anchor of the MNs.^[^ ^13^ ^]^ Another effective method to enhance the penetration of HFMs is cryopreservation.^[^ ^14^ ^]^ However, the freezing process is cumbersome, and the application time of cryomicroneedles is short. Therefore, designing high‐strength hydrogels to improve the mechanical properties of hydrated HFMs will help simplify the post‐processing steps of MN preparation and improve drug utilization.

Over the past decades, researchers have been dedicated to developing novel high‐performance hydrogel materials. This endeavor has resulted in the development of a series of hydrogels with enhanced mechanical properties through the introduction of effective energy dissipation mechanisms and improvements in network homogeneity.^[^ ^15^ ^]^ These include double‐network hydrogels,^[^ ^16^ ^]^ nanocomposite hydrogels,^[^ ^17^ ^]^ and slide‐ring hydrogels.^[^ ^18^ ^]^ Acrylic polymers, due to the modifiable physical and chemical bonds, are commonly used in the preparation of high‐strength hydrogels.^[^ ^19^ ^]^ Notably, a series of robust acrylic hydrogels have been constructed based on the dipole‐dipole (D‐D) physical interaction among the cyano groups of acrylonitrile (AN) in our group, leading to a significant enhancement in mechanical performance.^[^ ^20^ ^]^ Therefore, these high‐strength hydrogels present a potential way for the development of HFMs with effective transdermal penetration in the hydrated state.

**Figure 1:**
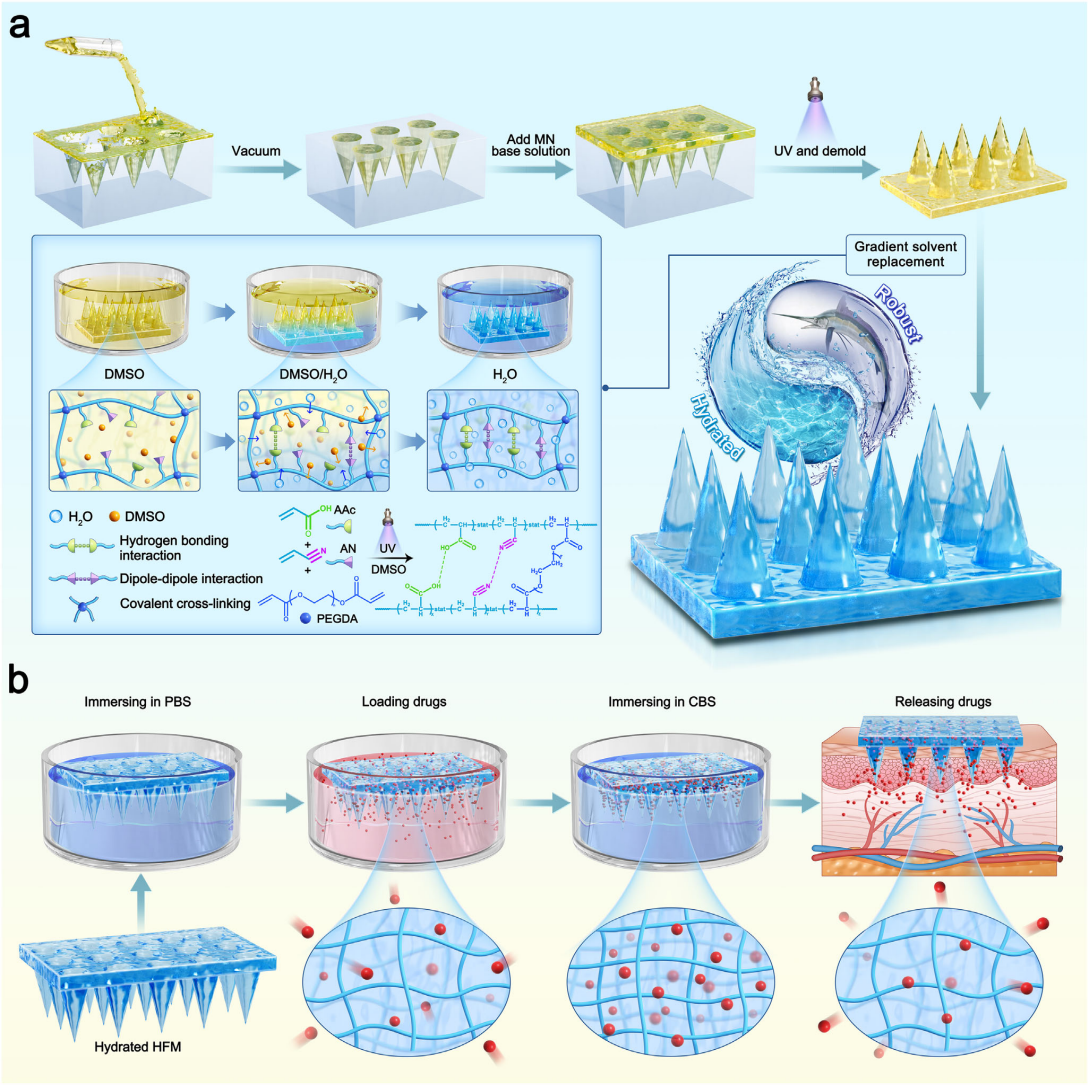
Schematic of the fabrication process for the AN/AAc‐MN patch and the procedure for drug loading and release. a) High‐strength hydrated AN/AAc‐MN is fabricated using the gradient solvent replacement method to establish the uniform formation of dipole‐dipole (D‐D) interactions and hydrogen bonding interactions. b) The pH responsiveness of AN/AAc‐MN realizes post‐drug loading capacity.

In this study, a pH‐responsive hydrated HFM for post‐drug loading, named AN/AAc‐MN, was developed by radical polymerization of AN, acrylic acid (AAc), and poly(ethylene glycol) diacrylate (PEGDA). Gradient solvent replacement was applied to promote the uniform formation of D‐D interactions and hydrogen bonding interactions, endowing the AN/AAc‐MN with sufficient mechanical properties to penetrate the skin in its hydrated state (**Figure** **1**a). In a phosphate buffer solution (PBS, pH 7.4), charge repulsion resulting from deprotonation of carboxyl groups weakens the D‐D interactions, thereby improving the entrapment of the drug into the loose hydrogel network. Upon re‐equilibration in a citrate buffer solution (CBS, pH 4) after gradient solvent replacement, the protonated carboxyl groups induce the cumulative re‐formation of D‐D interactions and hydrogen bonding interactions. The resultant tight network structure retains the drug inside, while strengthening the mechanical properties (Figure 1b). Upon application to the epidermis, the subcutaneous body fluid environment stimulates the prepared hydrated AN/AAc‐MNs to swell and release the drug. This post‐drug loading capacity, conferred by pH responsiveness, allows for the removal of unreacted monomers during MN fabrication, which not only improves the safety of the device but also prevents the problem of drug loss caused by traditional purification processes.^[^ ^21^ ^]^ Overall, the hydrated AN/AAc‐MN eliminates the adverse effects of the drying process, demonstrating potential as a minimally invasive transdermal drug delivery platform.

## 2 Results and Discussion

### 2.1 Design and Characterization of Hydrogels

In this work, to construct high‐strength pH‐responsive AN/AAc‐MNs suitable for post‐drug loading, AN was utilized as the primary monomer to enhance mechanical properties through the D‐D interactions between cyano groups. Additionally, AAc was selected as the secondary monomer to impart pH‐responsive behavior. PEGDA was used as a short‐chain crosslinker to enable chemical cross‐linking. The covalently cross‐linked hydrogel, named AN/AAc, was synthesized in two steps. First, a mixture of the monomers and the crosslinker in dimethyl sulfoxide (DMSO) was cured using UV light in the presence of the initiator lithium phenyl‐2,4,6‐trimethylbenzoylphosphinate (LAP), forming an organogel. The obtained organogel was then immersed in a series of solvent mixtures with different ratios of deionized water and DMSO for gradient solvent replacement. During this process, the hydrogen bond breaker DMSO within the gel network was gradually replaced by water molecules, facilitating the formation of uniform D‐D interactions and hydrogen bonding interactions between molecular chain segments.

The successful synthesis of the AN/AAc hydrogel was confirmed via Fourier‐transform infrared spectroscopy (FTIR) (Figure S1, Supporting Information). An absorption peak at 2242 cm^−1^ corresponds to the stretching vibration of C≡N groups.^[^ ^22^ ^]^ The peak appearing at 1728 cm^−1^ is attributed to C═O stretching in ester groups, while the signal from the anti‐symmetric stretching vibration of C─O─C in the PEGDA crosslinker is located at 1099 cm^−1^. The co‐existence of these functional groups and the disappearance of the C═C peak indicate successful covalent cross‐linking. The S═O Raman peak gradually red‐shifted and disappeared as the solvent replacement time increased (Figure S2, Supporting Information), indicating that the DMSO inside the hydrogel could be completely removed after a long period of solvent replacement.

**Figure 2:**
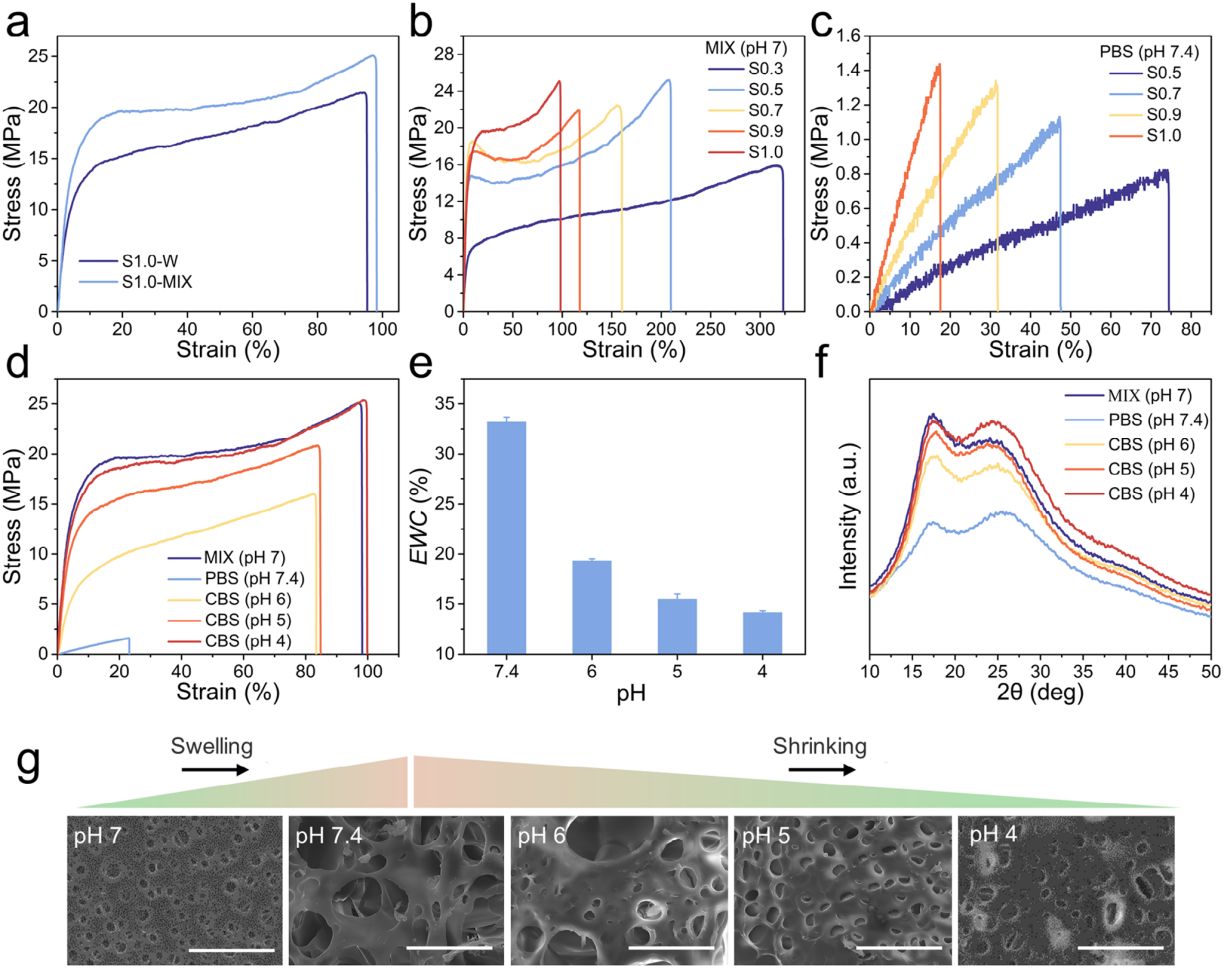
Mechanical properties and pH responsiveness of the AN/AAc hydrogel. Tensile stress‐strain curves of AN/AAc hydrogels are presented: a) fabricated using different solvent displacement methods (W, deionized water, MIX, mixed solution), b) with different solid contents after gradient solvent displacement, and c) with different solid contents equilibrated in PBS. d) Tensile stress‐strain curves, e) *EWC*, and f) XRD spectra of S1.0‐hydrogels equilibrated in different pH environments. g) SEM images of S1.0‐hydrogel at different pH values. Scale bars: 30 µm.

The mechanical properties of hydrogels obtained by different solvent replacement methods were subsequently investigated to evaluate the superiority of the gradient solvent replacement method (**Figure** **2**a). The hydrogel produced via the gradient solvent replacement method exhibited excellent tensile strength (*σ_b_*) of 25.86 ± 2.09 MPa, Young's modulus (*E*) of 407.35 ± 5.11 MPa, and breaking strain (*ε_b_*) of 101.20 ± 7.00%. This is attributed to its homogeneous and tightly physicochemical crosslinked structure. In contrast, the hydrogel formed by direct solvent replacement with deionized water showed relatively lower mechanical properties, with *σ_b_* of 21.14 ± 0.79 MPa, *E* of 205.13 ± 4.03 MPa, and *ε_b_* of 97.34 ± 5.28% (Figure S3a, Supporting Information). It is hypothesized that during the gradient solvent replacement, reversible physical bonds such as D‐D interactions and hydrogen bonding interactions have more time to align to form a homogeneous network structure. While direct solvent replacement with deionized water may lead to the rapid formation of local bonds, resulting in structural defects that impede stress transfer between the polymer chains and weaken the mechanical properties.^[^ ^15^ ^]^ Therefore, the hydrogel obtained through gradient solvent replacement possesses high stiffness, making it promising for the construction of hydrated‐state HFMs capable of transdermal drug delivery.

To maximize the deformation resistance of hydrated‐state hydrogels, tensile properties of the hydrogels were tested with varying solid contents after gradient solvent replacement. For convenience, the AN/AAc hydrogels were abbreviated as Sx, where x represents the ratio of initial monomer weight to total weight. After gradient solvent replacement, the S0.3 exhibited an *E* value of 111.59 ± 1.89 MPa (Figure 2b, and Figure S3b, Supporting Information). With an increase in solid content, the *E* value of the hydrogels rose above 400 MPa, while the *ε_b_* gradually decreased due to the high crosslink density. When these hydrogels were equilibrated in PBS, there was a significant reduction in mechanical properties due to the electrostatic repulsion of carboxylate ions formed by the deprotonation of carboxyl groups in the gel network, which disrupted the D‐D interactions and hydrogen bonds. As the solid content rose from 50 to 100 wt.%, the *σ_b_* and *E* values of the hydrogels equilibrated in PBS increased from 0.77 ± 0.05 MPa and 1.16 ± 0.01 MPa to 1.43 ± 0.01 MPa and 8.38 ± 0.08 MPa, respectively, while the *ε_b_* decreased from 83.6 ± 23.15% to 17.54 ± 0.18% (Figure 2c, and Figure S3c, Supporting Information). Notably, the S0.3 equilibrated in PBS was too weak and brittle to be tested. Considering the transdermal ability of the HFMs (*E* value of the hydrogel obtained via gradient solvent replacement) and the ability to resist skin pulling during HFMs drug release (*E* value of the hydrogel equilibrated in PBS), S1.0 was selected for subsequent experiments.

To explore the pH responsiveness, the pristine hydrogel equilibrated in deionized water was first soaked in PBS. Due to the deprotonation of carboxyl groups, the volume of the hydrogel expands, with an inverse proportion between volume expansion ratio and cross‐linking density (Figure S4, Supporting Information). Then, the hydrogels equilibrated in PBS were immersed in citrate buffer solutions (CBS) with different pH values until swelling equilibrium was reached. Given that the *pKa* value for acrylic acid is in the range of 4‐4.5,^[^ ^23^ ^]^ the pH values of CBS were adjusted to 4, 5, and 6, respectively. As the pH value of CBS decreased from 6 to 4, there was a gradual improvement in the *σ_b_*, *E*, and *ε_b_* of the corresponding hydrogels (Figure 2d). When the pH value of CBS exceeds the *pKa* of acrylic acid, the dissociated carboxylate ions remain in the network, thereby affecting the physical cross‐linking of the hydrogels. As the pH value of CBS equals the *pKa* of acrylic acid, the hydrogen bonds and D‐D interactions in the network are no longer affected by pH value, resulting in optimal mechanical properties of the hydrogel. In addition, the equilibrium water content (*EWC*) of the hydrogel networks equilibrated in CBS ranged from 14.17 ± 0.16% to 33.20 ± 0.48% with the increase of the pH values (Figure 2e), demonstrating a negative correlation between the solution pH value and the network cross‐linking density.

The micro‐morphology of the hydrogel network was further evaluated through scanning electron microscopy (SEM) to confirm the changes in the cross‐linking density. The prepared hydrogel with microporous structure showed swelling or shrinkage in response to different environmental pH values (Figure 2g). The pristine hydrogel equilibrated in deionized water (pH 7) displayed a relatively small microporous structure. When immersed in PBS, there was a notable increase in the pore size because of the decrease in crosslink density. Further immersing the hydrogels successively into CBS with decreasing pH values, the pore size gradually reduced, suggesting the reversible pH‐responsiveness.

The crystallization of the hydrogel was investigated through X‐ray diffraction (XRD) to further verify the effect of pH on the physical cross‐linking (Figure 2f). As the pH decreased from 7.4 to 4, there was a notable increase in the intensity of the sharp peak at 2θ = 17°, which is indicative of the crystallinity and molecular alignment of the polymer chains. For polyacrylonitrile‐based polymers, this sharp peak represents an ordered structure formed based on D‐D interactions.^[^ ^24^ ^]^ As the pH decreases, the protonation of the carboxylate ions induces a reorganization of the molecular structure, reinforcing the D‐D interactions and thus the ordered phase within polymer chains. In the CBS (pH 4) environment (below the *pKa* value of acrylic acid), the carboxylate ions are fully protonated to form carboxyl groups. The peak intensity recovered to a level similar to that of the MIX (pH 7) sample, indicating re‐formation of the homogeneous and tightly physicochemical crosslinked structure. Owing to the reversible pH‐responsiveness, these hydrogels hold promise for constructing HFMs with reproducible drug loading and release capabilities.

### 2.2 Fabrication and Characterization of AN/AAc‐MNs

**Figure 3:**
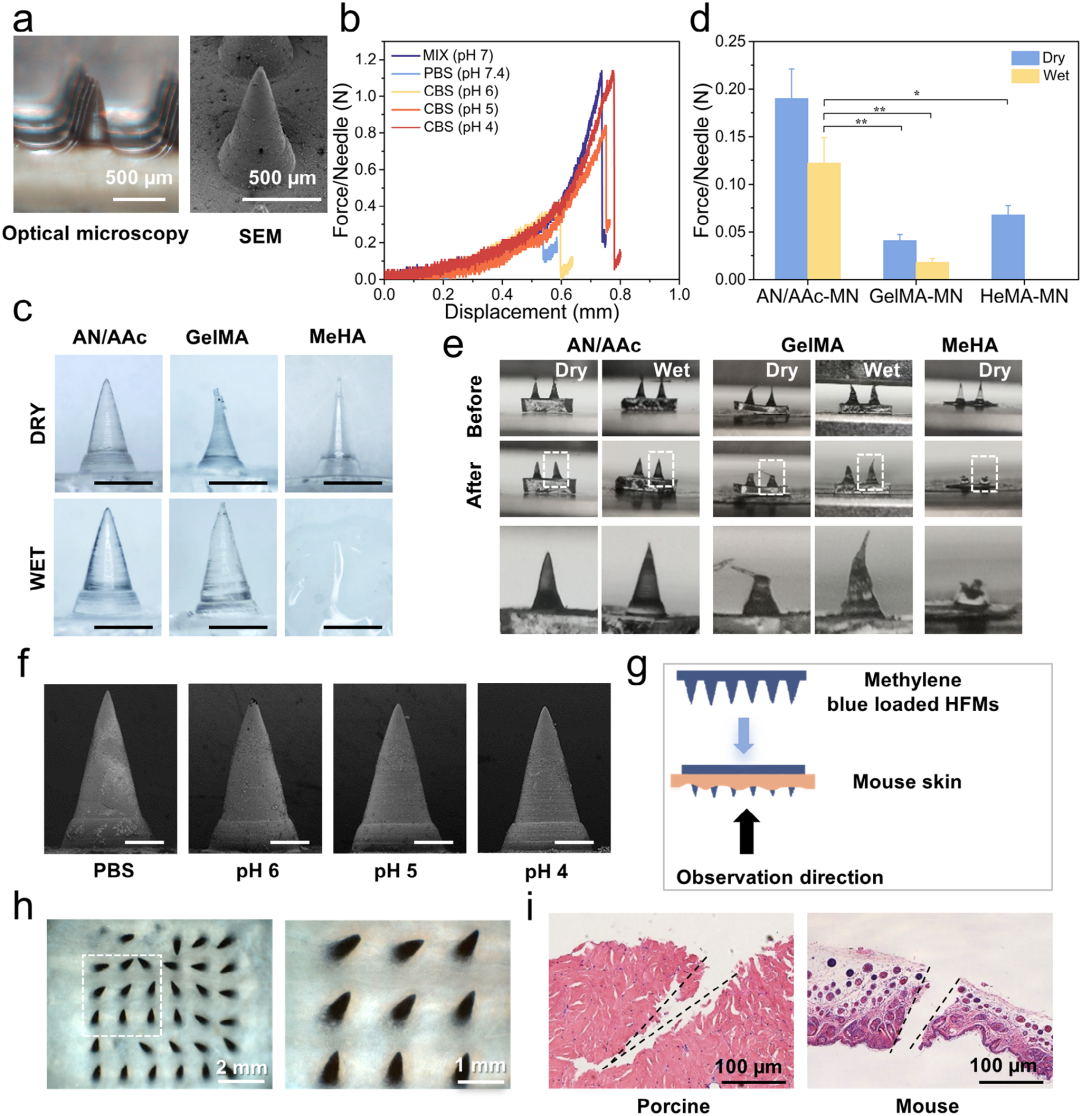
Morphology, mechanical properties, and skin penetration efficiency of the AN/AAc‐MNs. a) Morphology of the AN/AAc‐MNs imaged by optical microscopy and SEM. b) Mechanical behavior of AN/AAc‐MNs at different pH values. c) Morphological changes of different HFMs (AN/AAc‐MNs, GelMA‐MNs, and MeHA‐MNs) in dry and wet states. Scale bars: 500 µm. Comparison of the d) mechanical behavior and e) morphological changes of different HFMs after compression. f) The morphological changes of AN/AAc‐MNs at different pH values. Scale bars: 200 µm. g) Schematic illustration and h) gross picture of the AN/AAc‐MNs penetrating nude mouse skin in vitro. i) H&E staining of porcine and mouse skin punctured by AN/AAc‐MNs.

After exploring of the key properties of the AN/AAc hydrogels, AN/AAc‐MNs were fabricated using a template‐based approach with AN/AAc hydrogels, as illustrated in Figure 1a. First, the gel precursor solution containing AN, AAc, PEGDA and LAP was filled into the polydimethylsiloxane (PDMS) mold containing an array of MNs cavities (1000 µm height, 450 µm bottom diameter, 10–15 µm tip diameter, 1000 µm tip‐to‐tip distance) by vacuum processing, and then exposed to UV light for 20 min to ensure complete cross‐linking. Subsequently, the obtained organic AN/AAc‐MN was converted to a high‐strength hydrated AN/AAc‐MN via gradient solvent replacement. The hydrated AN/AAc‐MN array consisted of 6 × 6 cone‐shaped needles on a 9 × 9 mm substrate patch. Each needle was ≈722.50 ± 10.32 µm in height, with a 467.78 ± 1.92 µm bottom diameter, an 18.25 ± 4.18 µm tip diameter, and a 934.44 ± 13.47 µm tip‐to‐tip distance. The differences in these parameters compared with those of the PDMS mold can be attributed to the shrinkage of the organic AN/AAc‐MN after gradient solvent replacement. The morphology of AN/AAc‐MN was further evidenced by optical and SEM images (**Figure** **3**a). The sharp tips of the needles suggested the potential for AN/AAc‐MNs to puncture the stratum corneum. Upon applying an axial compression load to the AN/AAc‐MN, the failure force reached up to 1.17 ± 0.03 N per needle (Figure 3b), demonstrating sufficient mechanical properties for skin penetration.^[^ ^25^ ^]^ After equilibration in PBS, the failure force decreased to 0.47 ± 0.06 N per needle. As the buffer pH was gradually reduced to 4, the failure force of AN/AAc‐MN increased again to 1.15 ± 0.82 N per needle, indicating that the transdermal ability is reversibly pH‐responsive.

To establish a benchmark for direct comparison, the mechanical strength of other MNs fabricated by commonly used materials was also measured, such as gelatin methacryloyl (GelMA‐MN) and methacrylated hyaluronic acid (MeHA‐MN).^[^ ^13^, ^26^ ^]^ As shown in Figure 3c, the volume of the AN/AAc‐MNs exhibited minimal variation in both dry and wet states, with their needle tips remaining sharp and intact. In contrast, the needle tips of GelMA‐MN and MeHA‐MN underwent significant changes in the hydrated state. For example, MeHA‐MNs could not even maintain the original MN morphology, indicating high susceptibility to moisture. The force‐displacement curve showed that AN/AAc‐MNs had greater single needle mechanical force than those of GelMA‐MN and MeHA‐MN (Figure S5, Supporting Information). In the displacement range of 0–0.3 mm, the mechanical properties of dry AN/AAc‐MNs were the best, with a force of 0.18 ± 0.03 N per needle. The hydrated AN/AAc‐MNs were slightly weaker, with a force of 0.12 ± 0.03 N per needle, but still higher than GelMA‐MNs and MeHA‐MNs in both the dry and hydrated states (Figure 3d). Optical images of different HFMs before and after the compressive mechanics experiment were observed to investigate the morphology change in the MN shape (Figure 3e). Both dry and hydrated AN/AAc‐MNs maintained their MN shape relatively intact after compression. In contrast, the tips of the dry GelMA‐MNs, hydrated GelMA‐MNs, and dry MeHA‐MNs were notably damaged. This suggests that AN/AAc‐MNs possess superior mechanical properties compared to HFMs fabricated by the commonly used natural polymers.

It is worth mentioning that AN/AAc‐MNs are also pH‐responsive. The higher pH value led to an increased density of electrostatic repulsion between carboxylate anions, causing volume variations in the AN/AAc‐MNs. As shown in Figure 3f, the AN/AAc‐MN equilibrated at pH 4 displayed the lowest height of 743.36 ± 5.40 µm, whereas the AN/AAc‐MN swollen in PBS presented a height of 826.38 ± 1.83 µm. This reversible volume change in response to pH facilitates post‐drug loading of AN/AAc‐MNs and drug release in body fluid environments.

To verify the transdermal ability, AN/AAc‐MNs equilibrated at pH 4 were used to puncture isolated nude mice skin (Figure 3g). Before the experiment, the AN/AAc‐MNs were stained with methylene blue to facilitate visualization of the needle tips through the skin. As shown in Figure 3h, 91% of the needle tips completely penetrated the skin without fracture, indicating that the mechanical strength of the hydrated AN/AAc‐MNs is sufficient for transdermal administration. The hematoxylin and eosin (H&E) staining of the mouse skin and pig skin after the hydrated AN/AAc‐MNs treatment clearly showed the corresponding pinholes, indicating potential for deep drug delivery (Figure 3i). The dehydration step affected the morphology of the thin mouse skin, resulting in shorter pinholes in the stained sections than the length of AN/AAc‐MNs.^[^ ^27^ ^]^

### 2.3 In Vitro Drug Loading and Release Study

**Figure 4:**
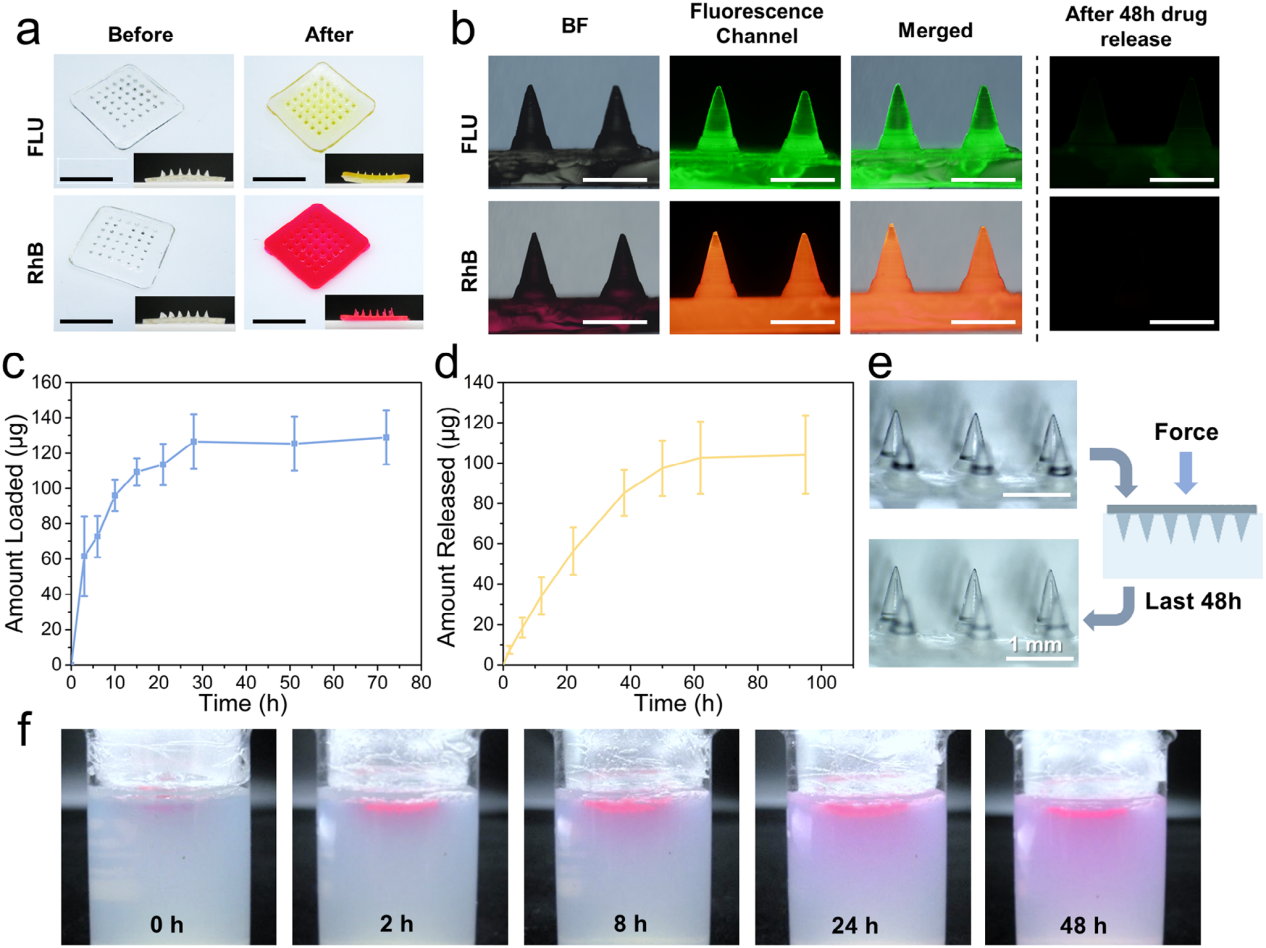
Drug loading and release properties of the AN/AAc‐MNs. a) Optical images of AN/AAc‐MNs before and after the loading of sodium fluorescein (FLU) and Rhodamine B (RhB). Scale bars: 1 cm. b) Representative bright‐field and fluorescence images of AN/AAc‐MNs loaded with FLU and RhB, and fluorescence images after 48 h drug release. Scale bars: 500 µm. c) The quantification of FLU loaded in AN/AAc‐MNs and d) the cumulative release rate curve of FLU from AN/AAc‐MNs within 100 h. e) Morphology of AN/AAc‐MNs before and after insertion into 2% (w/v) agar gel. Scale bars: 1 mm. f) In vitro drug release simulation of the RhB‐loaded AN/AAc‐MN.

To facilitate observation, the post‐drug loading capacity of the AN/AAc‐MNs was examined using sodium fluorescein (FLU) and Rhodamine B (RhB) as model drugs. Notably, the pH responsiveness of the AN/AAc‐MNs is based on carboxyl groups, which makes the system selective for the encapsulated drugs. Drugs such as doxorubicin, which are positively charged, can bind to the carboxylate groups through electrostatic interactions. Although the drug loading capacity is significantly improved, the drug release rate is too slow. As shown in **Figure** **4**a, the AN/AAc‐MNs equilibrated in PBS could absorb the drugs due to osmotic pressure differences. Upon re‐equilibration in a pH 4 environment to restore mechanical strength, the drugs remained inside the AN/AAc‐MNs. The fluorescence images demonstrated that FLU and RhB were uniformly distributed in the AN/AAc‐MNs (Figure 4b). Then, FLU was used to quantitatively evaluate the drug loading capacity of the AN/AAc‐MNs in post‐loading mode. The AN/AAc‐MNs equilibrated in PBS were immersed in the FLU solution, and the amount of loaded drug was determined by fitting with the standard curve (Figure S6, Supporting Information). It is obvious that an immersion time of 24 h is sufficient for the AN/AAc‐MNs to achieve complete drug loading, with a loading capacity of 128.85 ± 15.23 µg FLU for each AN/AAc‐MN patch (Figure 4c). In general, the pre‐loading mode of dissolving MNs is more controllable and requires only a homogeneous mixing of the drug into the MN tips, negating the need to dissolve the drug in solvents. However, the small volume of the dissolvable tip portion results in a limited amount of embedded drug and rapid drug release, thus requiring integration with drug reservoirs to prolong release.^[^ ^28^ ^]^ In contrast, the substrate of the AN/AAc‐MN formed in post‐loading mode acts as a drug reservoir, which can continuously penetrate drugs into the skin through the needle tips to achieve long‐term drug delivery.^[^ ^29^ ^]^

The drug‐loaded AN/AAc‐MNs were re‐equilibrated in a pH 4 environment to form strongly hydrated AN/AAc‐MNs for transdermal delivery. During this process, some drug loss is inevitable. Totally, ≈17.20 ± 2.23 µg encapsulated FLU was lost (Figure S7, Supporting Information). The in vitro drug release ability of the obtained strongly hydrated AN/AAc‐MNs was then tested. Both FLU‐loaded and RhB‐loaded AN/AAc‐MNs were each immersed in 5 mL PBS to facilitate swelling and drug release. As depicted in Figure 4b, the fluorescence intensity of the AN/AAc‐MNs significantly weakened over time, demonstrating the successful loading and release of both FLU and RhB. Quantitative analysis showed that 104.20 ± 19.36 µg FLU was sustainably released from the AN/AAc‐MNs within 48 h (Figure 4d), indicating that AN/AAc‐MNs could effectively release drugs. In addition, the tip morphology of the AN/AAc‐MN did not change significantly before and after puncturing the agar hydrogel, indicating that the AN/AAc‐MN is sufficiently stable to be completely removed without leaving any residue after application to the skin (Figure 4e). For real‐time observation, the RhB‐loaded AN/AAc‐MN patch was applied to a translucent agar hydrogel (2% w/v) under constant pressure to visualize the release profiles. As shown in Figure 4f, the red‐stained area in the agar hydrogel expanded over time, demonstrating that the pre‐loaded RhB could be released slowly and continuously.

### 2.4 In Vivo Drug Release Study and Biosafety Evaluation

**Figure 5:**
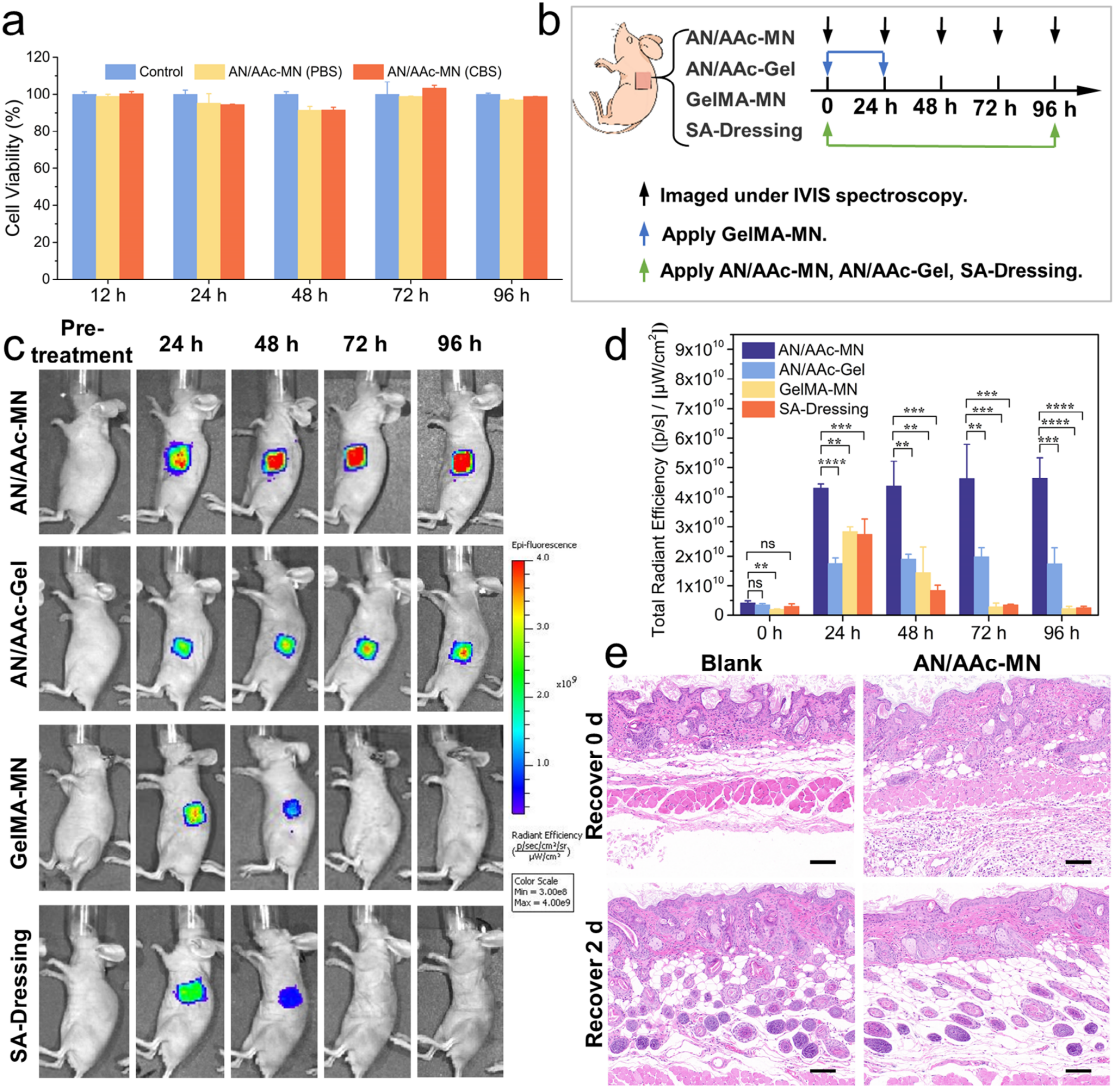
Biosafety evaluation and in vivo drug delivery of the AN/AAc‐MNs. a) Schematic showing the in vitro cytotoxicity test. b) Biocompatibility of the AN/AAc‐MNs assessed by CCK‐8 using L929 cells treated with extracts of AN/AAc‐MNs equilibrated in PBS and CBS. c) In vivo fluorescent imaging of mice treated with different MNs or dressing loaded with RhB at different time points. d) Quantification of fluorescence intensity on the treated skin. e) Skin sections in different states were stained by hematoxylin and eosin (H&E) staining. Recover 0 d: After 4 days of AN/AAc‐MN treatment and removal. Recover 2 d: Self‐recovery for 2 days. Blank: Normal skin without any treatment. Scale bars: 100 µm.

Biosafety is a critical consideration for the application of MNs. To evaluate the skin puncture recovery ability, a strong hydrated AN/AAc‐MN was applied on the flank skin of the mice for a duration of 5 min. The AN/AAc‐MN attached tightly to the skin without falling off. Compared with the untreated mice skin, there was a clear 6 × 6 array of pinholes at the treated site immediately after patch removal (Figure S8a, Supporting Information). The skin nearly fully reverted to its normal state within 60 min, without any obvious irritating reactions or permanent trauma. Furthermore, the biocompatibility of AN/AAc‐MNs was assessed through cytotoxicity testing. As shown in **Figure** **5**a, the viabilities of L929 cells treated with extracts of AN/AAc‐MNs equilibrated in CBS (pH 4) and PBS were both higher than 90%, indicating that the strong hydrated AN/AAc‐MNs have excellent biocompatibility throughout the entire process from initial application to pH‐responsive drug release.

To further investigate the sustained drug release capacity of the drug‐loaded hydrated AN/AAc‐MNs, an IVIS was employed to visualize the distribution of the drug within mice. The fluorescence intensity at the treated area exhibited a positive correlation with the concentration of RhB, thereby enabling the assessment of delivery and diffusion efficiency by comparing fluorescence intensities. As illustrated in Figure 5b, mouse skin was treated with different groups and then fixed with medical tape. The fluorescence intensity at the treated area was monitored at intervals of 24, 48, 72, and 96 h. The fluorescence intensity increased in all groups during the first 24 h, demonstrating the release and penetration of RhB (Figure 5c). The intensity in the AN/AAc‐MN group reached 4.31 × 10^10 [p/s]/[µW cm^−2^], which was significantly higher than that of the AN/AAc‐Gel group (Figure 5d). These results demonstrated the successful transdermal penetration of AN/AAc‐MN, which subsequently accelerated drug penetration.

Fluorescence images taken at subsequent time points revealed a gradual increase in fluorescence intensity in the AN/AAc‐MN group, rising from 4.19 × 10^9 to 4.64 × 10^10 [p/s]/[µW cm^−2^]. This indicates that RhB in the AN/AAc‐MN can be continuously released, accumulated, and diffused under the action of tissue fluid for more than 96 h. In future studies targeting specific chronic diseases, extended drug release capability of over weeks or months should be considered to assess its long‐term delivery potential. While the luminescence area and fluorescence intensity of the sodium alginate dressing group reached their peak at 24 h, they then gradually decreased with time until they were virtually non‐existent at 72 h. In vitro drug release experiment (Figure 4b) demonstrated complete RhB release from AN/AAc‐MNs within 48 h, which were completely immersed in PBS. In vivo, however, only the AN/AAc‐MNs tips that contacted tissue fluids resulted in prolonged drug release. The long‐term and sustained drug release of the AN/AAc‐MN group was attributed to the large drug loading capacity provided by the post‐loading mode, with the substrate acting as a reservoir. In addition, the AN/AAc‐MNs tips maintained their integrity after swelling, preserving the drug delivery microchannel and enabling continuous drug release upon stimulation by tissue fluids. It is worth mentioning that the tips of the GelMA‐MN group swelled and ruptured when peeled off at 24 h, making subsequent drug release testing unfeasible. The significant reduction in mechanical properties after swelling poses challenges for HFM constructed from natural polymers to achieve sustained drug delivery. The biosafety of the long‐term application was evaluated by the state of the skin surface after the unloaded AN/AAc‐MN was removed. Clear pinholes could be seen on the smooth skin surface 4 days after AN/AAc‐MN treatment (Figure S8b, Supporting Information). The skin returned to normal condition after 1 day without any obvious irritating reactions. After 2 days of recovery, there was still no inflammation, such as redness or swelling. Compared with untreated skin, some infiltrating inflammatory cells were observed in the skin sections treated with AN/AAc‐MN for 4 days (Figure 5e). After 2 days of self‐recovery, the inflammatory cells in the skin significantly reduced, indicating that the inflammatory response caused by AN/AAc‐MN application over a 4‐day period was within an acceptable range. All the results suggest that the prepared AN/AAc‐MN system can achieve long‐lasting (>4 days) transdermal drug delivery in a hydrated state.

## 3 Conclusion

In summary, a pH‐responsive, high‐strength AN/AAc‐MN was constructed using the gradient solvent replacement method. The AN/AAc system was designed to incorporate hydrogen bonds and D‐D interactions, thereby facilitating the formation of a uniform and dense network structure through gradient solvent exchange. This design enables the HFMs to maintain sufficient skin penetration strength even in a hydrated state. The properties of AN/AAc‐MNs, including mechanical properties, skin penetration capabilities, drug loading and release abilities, and biosafety, were thoroughly investigated. The hydrated AN/AAc‐MN achieved a transdermal strength of 1.15 ± 0.82 N, demonstrating effective skin penetration. Compared to traditional treatments at the same drug dose, AN/AAc‐MN showed superior drug delivery efficiency and sustained drug release ability for at least 4 days, outperforming conventional dressings and HFMs. These hydrated AN/AAc‐MNs with improved mechanical properties will simplify the storage conditions, making it a promising minimally invasive transdermal delivery platform. Looking ahead, future endeavors should aim at optimizing stimuli‐responsive polymer systems to accommodate a wider variety of drugs.

## 4 Experimental Section

### Materials

Trisodium citrate, citric acid, acrylic acid (AAc), acrylonitrile (AN), lithium phenyl (2,4,6‐trimethylbenzoyl) phosphinate (LAP), sodium fluorescein (FLU), and methylene blue were purchased from Macklin Chemical Reagent Company (China). Poly(ethylene glycol)diacrylate (PEGDA, Mn = 575) was purchased from Sigma–Aldrich Company (USA). Dimethyl sulfoxide (DMSO) was purchased from Shanghai Lingfeng Company (China). Phosphate buffer solution (PBS) was purchased from Beijing Solarbio Science & Technology Company (China). Rhodamine B (RhB) and sodium alginate were purchased from Aladdin Chemical Reagent Company (China). Agarose was purchased from Shanghai Baygene Biotechnologies Company (China). Isoflurane was provided by Tianjin Ruipu Biotechnology Company (China). Cell Counting Kit (CCK‐8) was obtained from Yeasen Biotechnology Company (China). Deionized water used in all experiments was purified using a Milli‐Q water purification system (Millipore Corporation, USA). Tegaderm film was purchased from 3M company (USA), while medical tape was purchased from HAINUO Company (China).

### Fabrication and Characterization of Hydrogels

First, AN, AAc, PEGDA575 and LAP were added to DMSO and vortexed for 10 min until completely dissolved to prepare the pre‐gel solution. Then, the solution was transferred into a homemade square plastic mold (side length 10 cm, thickness 0.5 mm) and irradiated with UV light for 30 min until complete polymerization. The obtained organogel was immersed in the mixed solution (DMSO: H_2_O = 3:1) for 12 h. Then, the sample was taken out and immersed in the mixed solution (DMSO: H_2_O = 1:1) for another 12 h. Finally, the gel was placed in deionized water for another 3 days to remove any residual DMSO and form a hydrogel. The deionized water was refreshed every 12 h.

Following the above method, hydrogels were prepared with a range of solid content. The formulations are further detailed in Table S1 (Supporting Information).

The mechanical properties of the hydrogels were measured using an in situ mechanical testing instrument (IBTC‐300SL, CARE Measurement and Control, China). Before the test, the hydrogels reached the equilibrium swelling state in the corresponding solutions and were cut into standard dumbbell specimens (width 2 mm, length 12 mm). The nominal strain and stress of the hydrogel samples were measured. The applied stretch speed was fixed at 50 mm min^−1^, and at least three specimens were tested.

To calculate the equilibrium water content (*EWC*) of the hydrogels in different pH values, the hydrogels were placed in the corresponding buffer for 3 days for full swelling, with the buffer refreshed every 12 h. The mass of the swollen hydrogels was weighed immediately after removing the surface water with filter papers. Then, the swollen hydrogels were placed into an oven at 60 °C for 2 days until the mass remained unchanged. *EWC* was calculated by the following equation:

(1) $$EWC\left(\%\right)=\frac{m_{0}-m_{d}}{m_{0}}\times 100\%$$

where *m_0_* was the mass of hydrogels equilibrated in the corresponding buffer, and *m_d_* was the dry mass of hydrogels.

To calculate the volume expansion ratio (*VER*) of hydrogels with different solid content (S0.5, S0.7, S0.9, and S1.0), the hydrogels first underwent gradient solvent replacement to reach swelling equilibrium in deionized water. Subsequently, they were immersed in PBS at 25 °C for 3 days, with PBS refreshed every 12 h. The *VER* was calculated by the following equation:

(2) $$VER\left(\%\right)=\frac{V_{1}-V_{0}}{V_{1}}\times 100\%$$

where *V_1_* was the volume of hydrogel equilibrated in PBS, and *V_0_* was the volume of hydrogel equilibrated in deionized water.

At the same time, scanning electron microscopy (SEM) (Phenom ProX G6, Thermo Fisher, USA) was used to observe the microstructure morphology of AN/AAc hydrogels in different pH environments. The AN/AAc hydrogels equilibrated at different pH values (pH 7.4, pH 7, pH 6, pH 5, pH 4) were freeze‐dried and sprayed with gold for observation.

The chemical structure of the hydrogel was characterized by Fourier transform infrared spectroscopy (FTIR, Nicolet 6700, Thermo Fisher, USA). The hydrogel (side length 2 cm, thickness 2 mm) was washed with water and completely dehydrated in 60 °C oven to obtain flat dried sample. FTIR spectra were collected in transmission mode from 4000 cm^−1^ to 400 cm^−1^. The DMSO content inside the hydrogel obtained after solvent replacement was verified by measuring the DMSO content in the replaced water. Raman spectra of the replaced water of the prepared S0.9 organogel at 12, 36, and 72 h were measured using LabRAM HR Evolution (Horiba, Japan). The laser power used was 20 mW, and the integration time was 25 s. X‐ray diffraction (XRD, D8 Advance, Bruker, Germany) measurements were performed to determine the phase structures of the hydrogels in different pH values (pH 7.4, pH 7, pH 6, pH 5, pH 4). The obtained data were typically collected by continuous scanning at a range of 5–50° with a scanning rate of 8°/min.

### Fabrication of AN/AAc‐MNs

AN/AAc‐MNs were fabricated by micro‐mold casting method. The pre‐gel solution prepared according to the composition of the S1.0‐hydrogels was distributed into the PDMS molds (36 conical needles (6 × 6) with 1000 µm needle height, 450 µm column width, 10–15 µm tip diameter, and 1000 µm interspacing). The molds were placed in a vacuum chamber at 0.07 MPa for 5 min to achieve degassing and fill the MN cavities with the solution. The solution was cast again until the substrate became completely flat. Then, the molds were exposed to the UV light (365 nm, 300 W) for 20 min to facilitate the curing of pre‐gel solution. The obtained organic samples were demolded and underwent gradient solvent replacement to remove the residual monomers and rebuild the AN/AAc‐MNs. Specifically, the demolded samples were first immersed in the mixed solution (DMSO: H_2_O = 3:1) for 12 h. Then, the samples were taken out and immersed in the mixed solution (DMSO: H_2_O = 1:1) for another 12 h. Finally, the obtained samples were placed in deionized water for 3 days to remove any residual DMSO. The deionized water was refreshed every 12 h.

Taking advantage of the pH responsiveness of the hydrogel, the drug was loaded into the AN/AAc‐MNs by diffusion. The fabricated blank AN/AAc‐MNs were immersed in PBS (pH 7.4) for 3 days to reach swelling equilibration. Then, the AN/AAc‐MNs were immersed in the drug solution for 12 h for drug loading. Finally, they were sequentially immersed in CBS with the pH value of pH 6, pH 5, pH 4 at an interval of 12 h to get the drug‐loaded AN/AAc‐MNs.

### Characterization of AN/AAc‐MNs

The detailed morphology of the AN/AAc‐MNs was analyzed by a stereomicroscope (RH‐2000, hirox, Japan) to investigate the sharpness of the tips and the arrays distribution. SEM was used to measure the structural parameters of the AN/AAc‐MNs.

The mechanical properties of the HFMs were measured using an in situ mechanical testing instrument (IBTC‐300SL, CARE Measurement and Control, China). The HFMs were pre‐cut into 1 × 1 tip, which were placed on the fixed platform with the tip facing upward. The force probe gradually compressed the HFM tips at the rate of 0.1 mm/min and stopped at a displacement of 1 mm. The mechanical properties of other commonly used MN materials were also measured. At least three specimens were tested in each set of the experiments.

To evaluate the skin penetration efficiency, the AN/AAc‐MNs dyed by methylene blue were inserted into the mouse skin by hand pressure, which was subsequently observed under a bright field microscope (Olympus BX53, Olympus Corporation, Japan). In addition, AN/AAc‐MNs were applied onto the porcine skin and mouse skin by thumb force for 5 min. Then, the penetrated skin was immersed in 4% paraformaldehyde solution, followed by embedding in paraffin sections and cryosection. The skin sections were stained with hematoxylin and eosin (H&E) for histological analysis.

### In Vitro Evaluation of HFMs

To test the drug loading efficiency, FLU and RhB were used as model drugs. The blank AN/AAc‐MNs equilibrated in PBS were immersed in 5 mL of the drug solution, which was made from PBS containing 100 µg mL^−1^ of FLU. At each determined time point (0, 0.5, 1, 3, 6, 9, 12, 24, 36, 48, 72 h), 200 µL of the solution was collected to a 96‐well plate for measurement of fluorescence intensity using a microplate reader (Varioskan LUX, Thermo Fisher, USA) (FLU: excitation 490 nm/emission 514 nm). While the same volume of drug solution was added back to the systems. The concentrations of the solution corresponding to each time point were quantified using the standard curves. The amount of drug loaded was then calculated using the following equation:

(3) $$M_{drug}=\left(C_{0}-C_{t}\right)\times 5$$

where *C_0_* was the initial concentration of the solution, *C_t_* was the concentration of the solution at each time point.

The AN/AAc‐MNs loaded with the model drugs were imaged using a Positive fluorescence microscope (Olympus BX53, Olympus Corporation, Japan), with excitation at 488 nm and emission at 493–634 nm.

To test the drug release efficiency, the drug‐loaded AN/AAc‐MNs were immersed in 5 mL PBS (pH 7.4). At the desired timepoints (0, 2, 6, 12, 24, 36, 48, 60, 72, 96 h), the same method was used to collect the release medium and measure the absorbance. The cumulative release amount and rate were analyzed.

Agarose gel (2% w/v) was prepared to simulate human skin. The RhB‐loaded AN/AAc‐MN was inserted into the gel, with a constant pressure applied above the substrate of the AN/AAc‐MN to ensure continuous penetration of the needle. Then, the actual release and diffusion of RhB at different time points (0, 2, 8, 24, 48 h) were observed. Additionally, the morphology of the AN/AAc‐MNs was photographed and analyzed before and after the drug release simulation.

### Biosafety Evaluation of AN/AAc‐MNs

In order to observe the recovery of the skin after the MN administration, the mice were depilated on their dorsal skin with depilatory cream 1 day in advance. Mice with smooth skin and no redness or swelling were selected for the experiment. For short‐term safety characterization, the AN/AAc‐MN was inserted into the dorsal skin with thumb pressure. After 5 min of application, the AN/AAc‐MN was removed, while the treated skin was photographed at certain intervals (0, 20, 40∖, 60 min) using a digital camera (IXUS 860 IS, Canon, Japan) to observe the recovery of the pinholes. For long‐term safety characterization, the AN/AAc‐MN was inserted into the dorsal skin with thumb pressure and fixed with medical tape for 4 days. After removing the AN/AAc‐MN, the treated skin was photographed at certain intervals (0, 1, 2 days) using IXUS 860 IS to observe the redness, swelling and recovery of the skin. The treated skins (0, 2 days) were collected and immersed in 4% paraformaldehyde solution, followed by embedding in paraffin sections and cryosections. The skin sections were stained with H&E for histological analysis.

The cytocompatibility of different AN/AAc‐MNs (equilibrated in PBS and in pH 4 CBS after post‐drug loading) on L929 fibroblast cell lines was analyzed by CCK‐8 assay. First, AN/AAc‐MNs were sterilized by UV irradiation for 48 h, and rinsed with sterile water. Then, AN/AAc‐MNs were incubated with DEME medium (0.1mg mL^−1^) containing 10% fetal bovine serum at 37 °C for 24 h. The obtained medium was diluted with DEME medium at a ratio of 1:1 to form extracts. L929 cells were seeded into 96‐well plate at a density of 5 × 10^4^ cells per well and incubated with the extract medium for 12, 24, 48, 72, or 96 h. After that, 10 µL of CCK8 solution was added to each well, and the cells were further cultured for 3 h. Finally, the absorbance was measured at 450 nm using a microplate reader. The cell viability was calculated according to the following equation:

(4) $$Cellability\left(\%\right)=\frac{C_{s}-C_{0}}{C_{c}-C_{0}}\times 100\%$$

where *C_s_* was the absorbance of the sample, *C_0_* was the absorbance of the blank group, and *C_c_* was the absorbance of the control group.

### In Vivo Drug Release

To evaluate the efficiency of the AN/AAc‐MNs in releasing drug, BALB/c nude female mice (8‐10 weeks old) were used in this study and were bought from Charles River Laboratory Animal Technology Corporation in Beijing (China). All of the animal experiments were performed according to the protocol approved by the Institutional Animal Care and Use Committee (IACUC) of Chinese Academy of Sciences, Shenzhen Institutes of Advanced Technology (SIAT‐IACUC‐230728‐YYS‐TXXZX‐ZXL‐A0727‐01). Small animal live imaging system (IVIS) (CALIPER IVIS SPECTRUM, CALIPER, USA) was used to visualize in vivo drug release. Nude mice were divided into 4 groups (n = 5). AN/AAc‐MNs, AN/AAc‐Gel, sodium alginate dressings, and GelMA‐MNs loaded with the same dose of RhB were applied to the flanks of the mice. The applied MNs were fixed with medical tape to ensure firm contact with the skin. The mice were anesthetized with isoflurane using a gas anesthesia. Fluorescence intensity of the treatment area was measured using the IVIS system before MN application and at 24, 48, 72, and 96 h post‐application. MNs were temporarily removed at each time point for imaging and re‐applied to the same puncture sites along the previously created needle holes on the skin, ensuring consistent re‐treatment for continued drug release. Living Image 4.0 was used to quantitatively analyze the fluorescence data.

### Statistical Analysis

All data were presented as mean ± standard deviation. All statistical calculations between two groups were analyzed by one‐tailed *t*‐test through GraphPad Prism 8.0.2. The difference was considered statistically significant when *p* < 0.05.

## Conflict of Interest

The authors declare no conflict of interest.

## Supporting information



Supporting Information

## Data Availability

The data that support the findings of this study are available from the corresponding author upon reasonable request.
