# Supplementary material for: Gradient Solvent Replacement‐Mediated Formation of High‐Strength Hydrogel‐Forming Microneedle for Long‐Term Drug Delivery
Source: Adv Sci (Weinh). 2025 May 2;12(30):2500833. doi: 10.1002/advs.202500833 (PMC12376595; doi:10.1002/advs.202500833)
Supplement: Supplementary file 1 — Supporting Information [file ADVS-12-2500833-s001.docx]

Supporting Information

Gradient Solvent Replacement-Mediated Formation of High-Strength Hydrogel-Forming Microneedle for Long-Term Drug Delivery

*Hui Li^1^, Fengzhen Meng^2^, Chengwei Hu^1,3^, Zhiyun Wu^1,3^, Liuzhi Hao^1,3^, Caijun Sun^4^, Lijing Fang^1^, Fan Pan^1^, Shaoquan Bian^1,3^, Huipeng Li^5^, Mingjun Li^5^, Bo Liu^5^*, Xiaoli Zhao^1,3^**

H. Li, C. Hu, Z. Wu, L. Hao, L. Fang, F. Pan, S. Bian, X. Zhao

^1^Institute of Biomedicine and Biotechnology

Shenzhen Institute of Advanced Technology

Chinese Academy of Sciences

Shenzhen 518055, PR China

E-mail: zhao.xl@siat.ac.cn

F. Meng

^2^Institute of clinical translation and regenerative medicine

People’s Hospital of Baoan District

The Second Affiliated Hospital of Shenzhen University

Shenzhen 518101, PR China

C. Hu, X. Wu, L. Hao, S. Bian, X. Zhao

^3^University of Chinese Academy of Sciences

Beijing 100049, PR China

C. Sun

^4^School of Public Health (Shenzhen)

Sun Yat-sen University

Shenzhen 518107, PR China

H. Li, M. Li, B. Liu

^5^Hebei Key Laboratory of Biomaterials and Smart Theranostics

School of Health Sciences and Biomedical Engineering

Hebei University of Technology

Tianjin 300130, PR China

E-mail: bo.liu@hebut.edu.cn


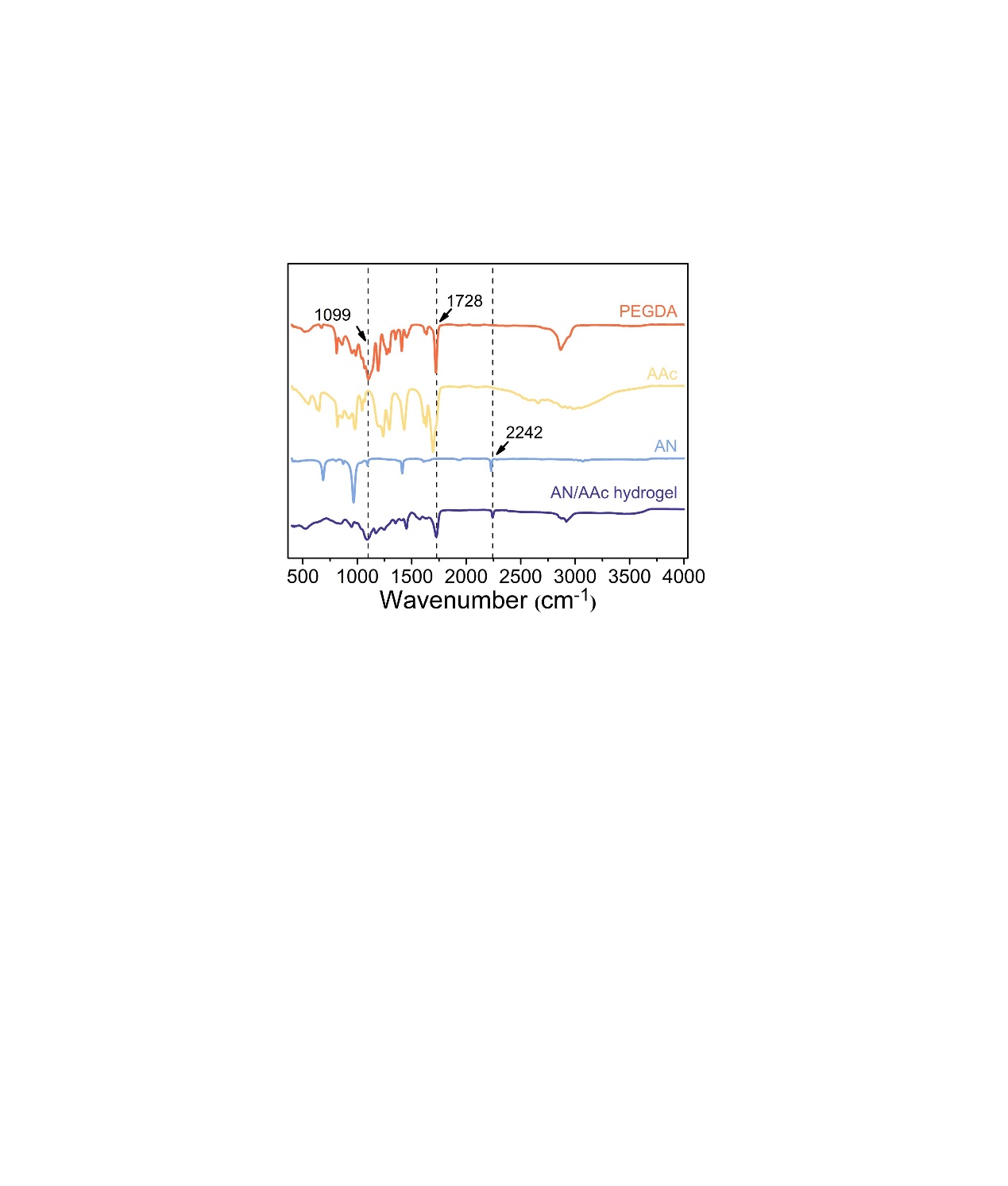


**Figure S1.** Chemical composition of the AN/AAc hydrogel. FTIR spectra of the prepared hydrogel and the component monomers.


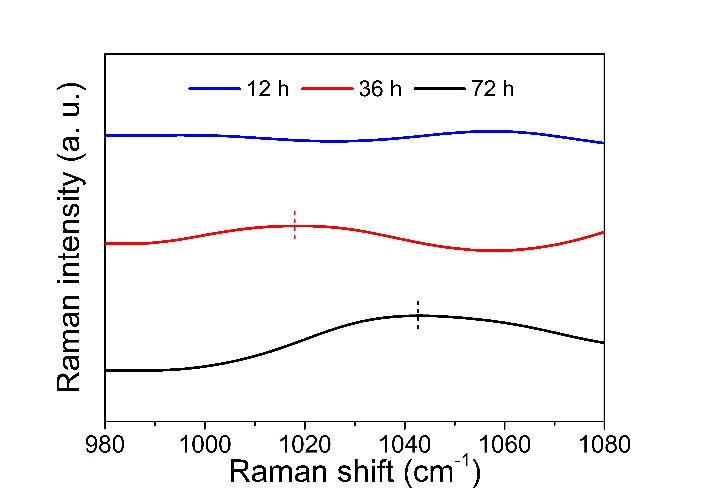


**Figure S2.** Residual DMSO in the hydrogel after solvent replacement. Raman spectra of the S=O group with different solvent replacement times.


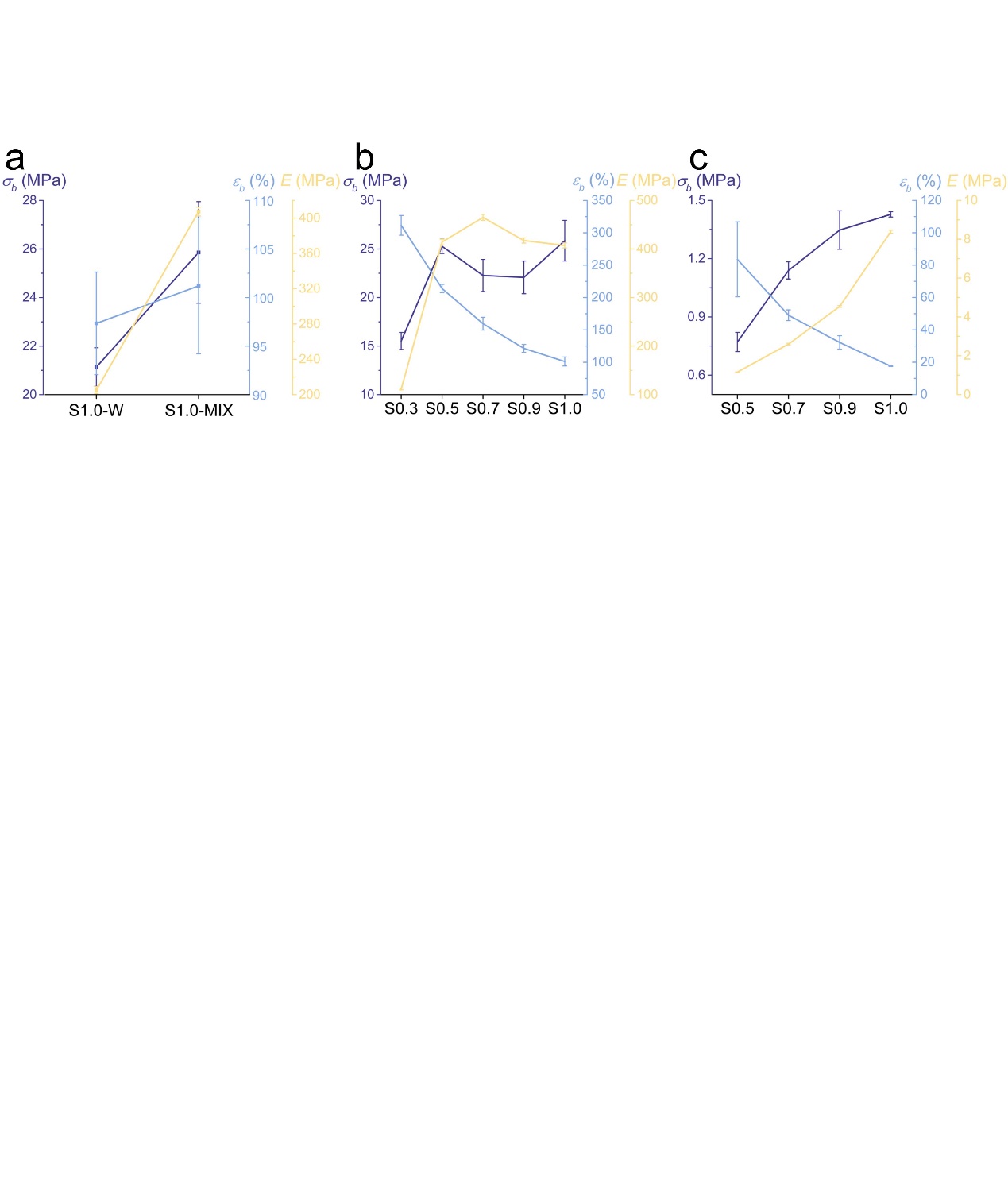


**Figure S3.** Mechanical properties of the AN/AAc hydrogel. (a) Breaking strain (𝜀_𝑏_), tensile strength (σ_𝑏_), and Young’s modulus (*E*) of the hydrogels made through different solvent replacement methods. (b) 𝜀_𝑏_, σ_𝑏_, and *E* of the hydrogels with different solid contents after gradient solvent replacement treatment. (c) 𝜀_𝑏_, σ_𝑏_, and *E* of the hydrogels with different solid contents after equilibration in PBS.


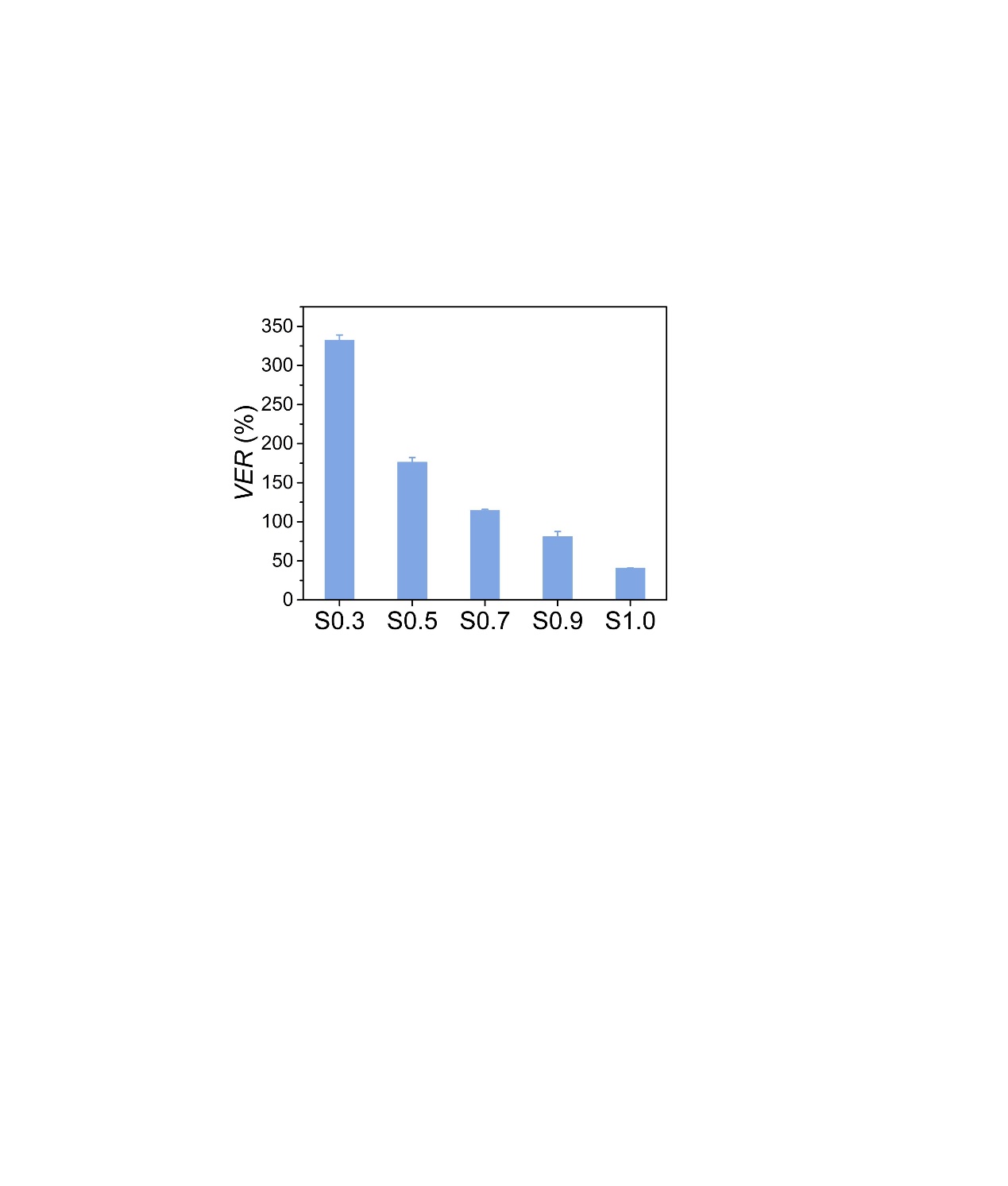


**Figure S4.** pH responsiveness of the AN/AAc hydrogel. Volume expansion ratio of the hydrogels with different solid content from gradient solvent replacement to PBS equilibrium.





**Figure S5.** Mechanical comparison of different HFMs. Compressive mechanical performance of different HFMs. The compression displacement was set to 0.3 mm.


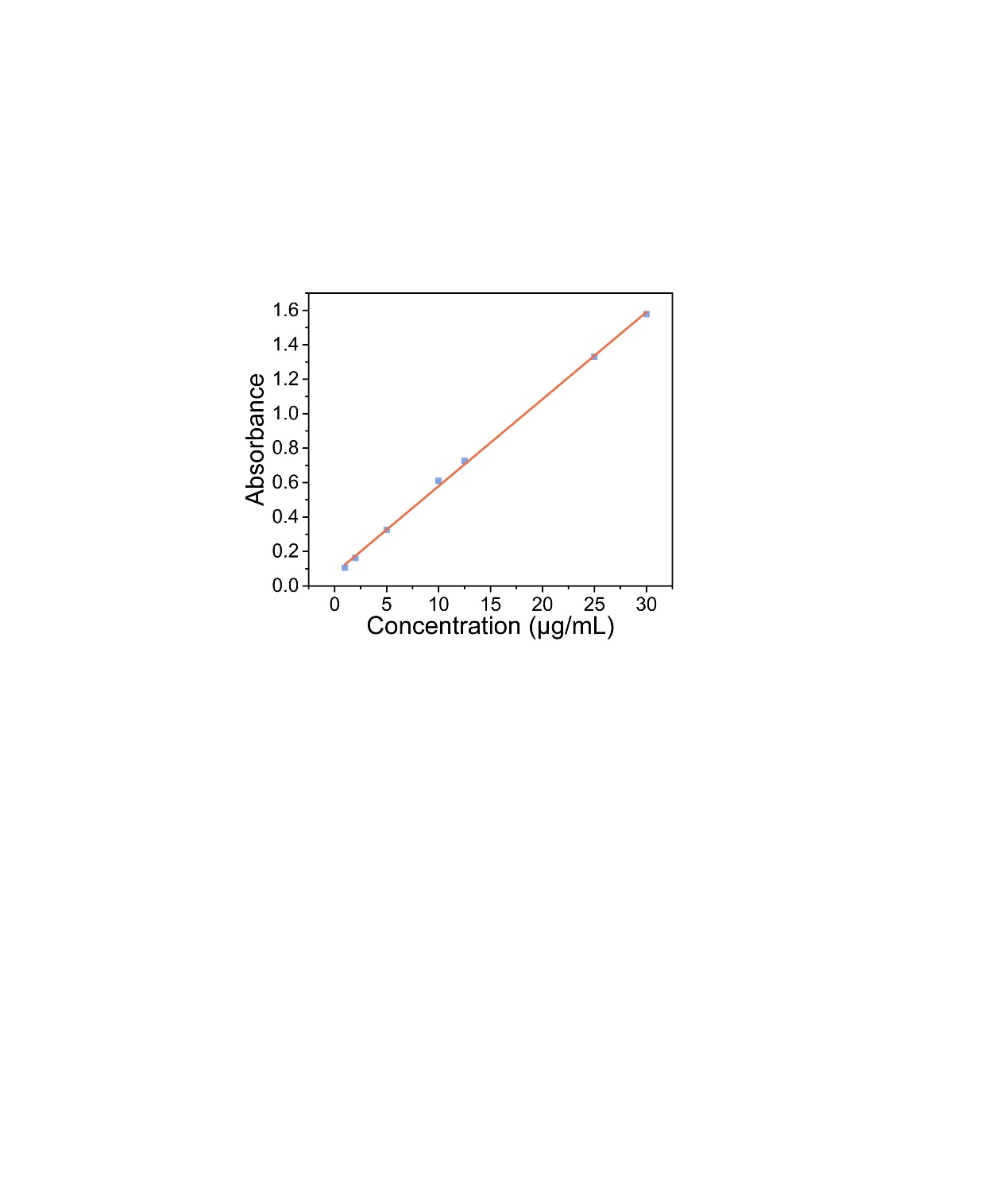


**Figure S6.** *In vitro* evaluation of the drug release of the AN/AAc-MNs. Standard curve of sodium fluorescein solution.


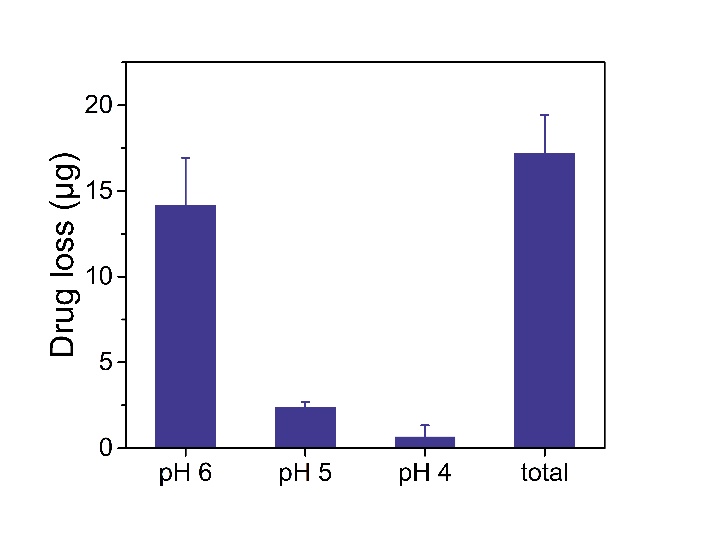


**Figure S7.** Drug loss during the acid soaking process of the AN/AAc-MNs. Drug loss in different CBS during the acid enhancement process of AN/AAc-MNs loaded with FLU (equilibrated in PBS).


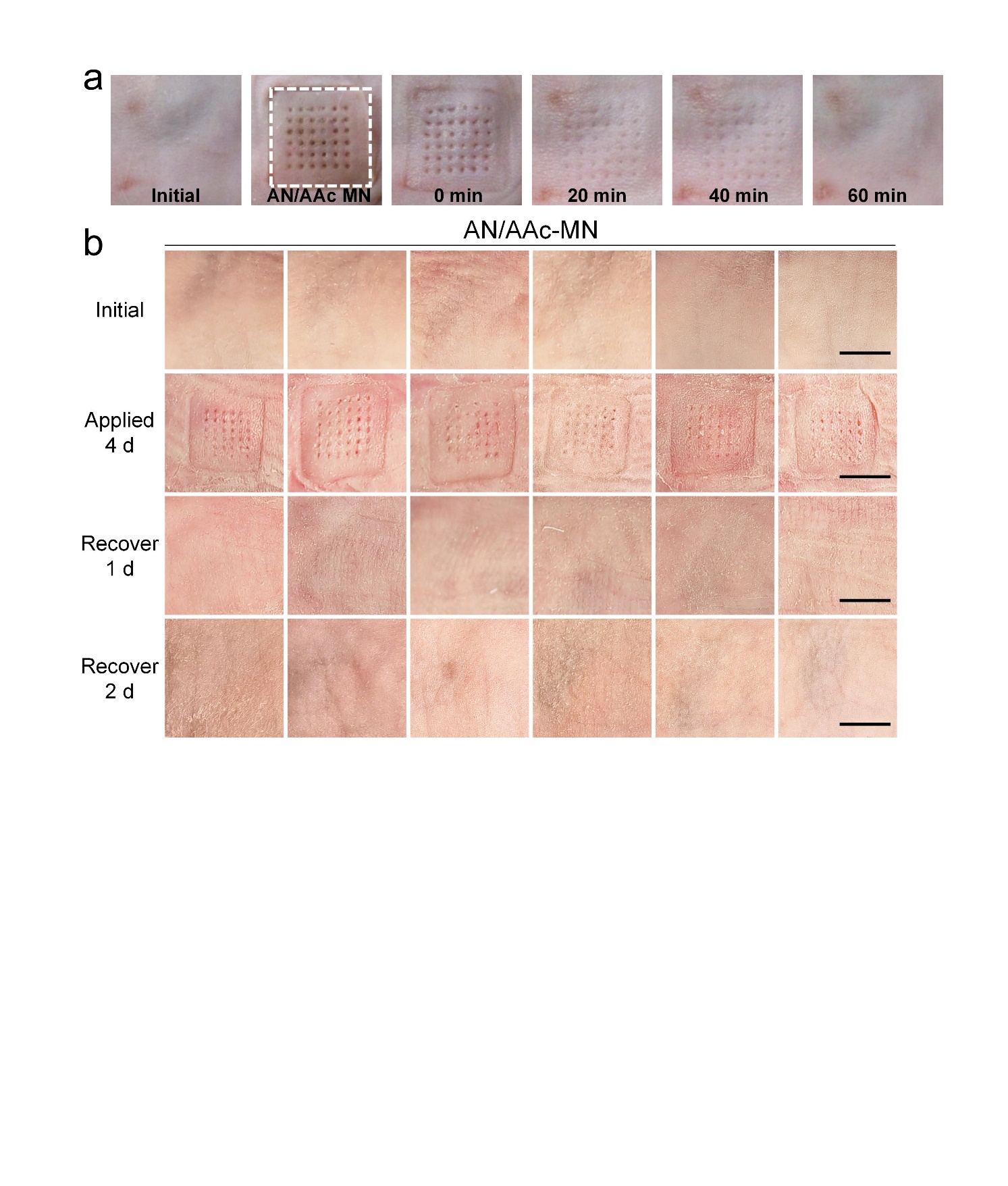


**Figure S8.** Biosafety of the AN/AAc-MN. (a) Recovery ability of the mouse skin treated with AN/AAc-MN for 5 min. (b) Photographic pictures of mice skins in different states. Initial: Before AN/AAc-MN treatment. Applied 4 d: After 4 days of AN/AAc-MN treatment and removal. Recover 1 d: Self-recovery for 1 day. Recover 2 d: Self-recovery for 2 days. Scale bars: 5 mm.

**Table S1.** Formulation for hydrogels with a range of solid content.

| Formulation code | Solid content (%) | AN  (mL) | AAc  (mL) | PEGDA575  (mL) | DMSO  (mL) | LAP  (mg) |
| --- | --- | --- | --- | --- | --- | --- |
| S0.5  S0.7  S0.9  S1.0 | 50  70  90  100 | 1.0  1.4  1.8  2.0 | 0.20  0.28  0.36  0.40 | 0.30  0.42  0.54  0.60 | 1.5  0.9  0.3  0.0 | 7.5  10.5  13.5  15.0 |
